# Rethinking non-urgent EMS conveyance to ED during night-time - a pilot study in Southwest Finland

**DOI:** 10.1186/s12873-023-00872-0

**Published:** 2023-08-23

**Authors:** Aleksi Kasvi, Timo Iirola, Hilla Nordquist

**Affiliations:** 1 Emergency Medical Services, Turku University Hospital and University of Turku, PO Box 52, Turku, 20521 Finland; 2 Department of Healthcare and Emergency Care, South-Eastern Finland University of Applied Sciences, Pääskysentie 1, Kotka, 48220 Finland

**Keywords:** Emergency medical services, Paramedic, Emergency department, After-hours care, Scope of practice, Pilot study, Emergency medical technician, Allied health

## Abstract

**Background:**

The amount of emergency medical service missions has increased internationally in recent years, and emergency departments are overcrowded globally. Previous evidence has shown that patients arriving at the emergency department during nighttime (20 − 08) have to wait longer, are more likely to leave without being seen, and often have non-urgent conditions compared to patients arriving during the day. The objective of this pilot study was to examine what kind of patient groups are conveyed as non-urgent to the hospital by emergency medical service during nighttime and what kind of diagnostic tests and medical interventions those patients receive before morning to identify patient groups that could be non-conveyed or directed to alternative points of care.

**Methods:**

This was a retrospective register study where the information of patients conveyed to university hospital during nighttime (20 − 08) were analyzed. Frequencies of the dispatch codes presenting complaints, medical treatments, and diagnostic tests were calculated. Age significance (under/over 70 years) was also tested.

**Results:**

73.5% of the patients received neither medical treatment nor had diagnostic tests taken before morning. Most of these were patients with mental disorder(s), hip pain/complaint, or laceration/cut. Almost half of the patients with abdominal pain or fever had laboratory tests taken. Patients over 70 years old received more medications and had more diagnostic tests taken than younger patients.

**Conclusions:**

Some of the low-acuity patients could be non-conveyed or referred to alternative pathways of care to avoid impolitic use of emergency medical service and to reduce the workload of emergency departments. Further research is needed to ensure patient safety for patients who are not conveyed at night.

## Background

The amount of Emergency Medical Service (EMS) missions has increased internationally during previous years [1–3], especially due to population aging [4, 5]. The role of being exclusively an emergency responder has evolved in pre-hospital emergency care, requiring current EMS personnel to have a holistic grasp of effectively managing both acute emergencies and non-emergency patient situations [5–7]. At the same time, Emergency Departments (EDs) are overcrowded globally [8]. Previous evidence has shown that patients arriving to ED during nighttime (20 − 08) have to wait longer, are more likely to leave without being seen, and often have non-urgent conditions compared to the patients arriving during the day [9]. In addition, some evidence suggests that approximately 5% of the patients leave the ED by themselves before treatment is completed [10].

All EMS patients do not have to be conveyed to the hospital but can be safely treated and left at the scene or referred to primary health care later. Non-conveyance saves time and EMS resources [11]. According to previous evidence, non-conveyance rates vary in different countries (11–56%) but also among EMS providers depending on the paramedics’ skill levels, management’s perceptions of non-conveyance [12, 13], and the level of collaboration between health and social care [12] and general practitioners [14]. EMS non-conveyance is found to be a relatively patient-safe method of avoiding ED crowding [15, 16].

As paramedics are capable of performing out-of-hospital triage [17–21], it would be beneficial for patients and the healthcare system to identify non-urgent patient groups that could be treated at home [5] or directed to alternative points of care instead of conveyance to the ED [21–24]. Previous study results have reflected that, for example, patients who have self-harmed themselves might benefit from alternative pathways of care [25], and patients with psychiatric complaints could be directed to specialty care after evaluation from an advanced level paramedic [26].

In Finland, the EDs of local hospitals in Loimaa and Uusikaupunki discontinued their services during nighttime in 2017 [27]. This may affect the safety and quality of the health services, as the distance to the nearest ED increased for the citizens who live within the sphere of influence of those hospitals. Consequently, the time per EMS mission increased as conveyance takes longer, and longer EMS mission times decreased the amount of free EMS resources in the area. However, certain patients could be directed to the local ED or primary health care the next morning to avoid unnecessary conveyance during nighttime.

Our pilot study examined what kind of patient groups are conveyed as non-urgent to ED by EMS during nighttime and what kind of laboratory tests, X-rays and medical interventions those patients receive before morning. Based on this pilot study, we deliberate if a more extensive register study is needed in order to identify the patients who could be non-conveyed during nighttime and directed to the local health services the next morning. Overall, the purpose is to develop EMS non-conveyance protocols and discuss the needs and possibilities for expanding paramedics’ scope of practice. A pilot study approach was selected as it is a suitable method for recognizing the patient groups which could benefit from alternative pathways of care and for focusing further studies of these patient groups [28].

## Methods

### Study design

We performed a retrospective study based on the patient registry of the Hospital District of Southwest Finland (since 2023, The Wellbeing Services County of Southwest Finland) from January 1st 2020 to December 31st 2020. Adult, non-urgent EMS patients that were conveyed from the Southwest Finland towns of Loimaa and Uusikaupunki by EMS to the university hospital ED during nighttime (20 − 08) were included in this study.

In Finland, EMS missions are divided into four different categories according to their urgency (A-D), with the most urgent (A and B) carried out as emergency missions with emergency response driving. The emergency centre first determines the urgency of the mission based on a risk assessment, after which the paramedics determine the urgency of the possible conveyance. This study focuses on patients conveyed as category D (non-urgent).

### Setting

At the time of this pilot study, the hospital district of Finland was responsible for organizing EMS. In the study area, Southwest Finland Emergency Services, Hospital District of Southwest Finland, and a private service provider performed EMS missions under the supervision of the hospital district [29]. There were 34 ambulances in the area and a physician-staffed helicopter EMS (HEMS) unit. The EMS units involved in this research are advanced level units, manned by one basic level paramedic with three-year vocational education and one advanced level paramedic or two of the latter. Advanced level paramedics are registered nurses with advanced-level out-of-hospital specialization or Emergency Care/Nursing dual Bachelor’s degree [6].

Both Loimaa (population 15,770) and Uusikaupunki (population 15,378) [30] are located around 70 km from the Turku University Hospital and have urban and rural areas. The ambulances are stationed in urban areas, and the usual conveyance time to the university hospital is approximately one hour without emergency response driving. The EDs of the local hospitals in Loimaa and Uusikaupunki are small units with one doctor. However, a comprehensive range of imaging, including computed tomography and laboratory tests are available. There are no doctors in the local hospitals outside the ED opening hours (08–20).

### Description of the data

The study material was collected in two phases. First, patients of EMS non-urgent (D) missions that began between 20 and 08 and resulted in conveyance to the university hospital were identified from the patient information system used by the EMS and their EMS dispatch codes and presenting complaints (International Classification of Primary Care, 2nd edition, ICPC-2) were collected. Only patients that were conveyed as non-urgent (D) were included in the study. Secondly, patient records and nursing reports of the patients identified in phase one were retrieved from the patient information system used in the Turku University Hospital using personal identification codes. Laboratory tests, x-rays, and medical interventions performed during nighttime (20–08) and the ED ICPC-2 codes of each patient were recorded.

### Statistical methods

First, the data collected from the EMS patient information system were organized, and the frequencies and percentages of different EMS dispatch codes and EMS ICPC-2 codes were calculated. Second, the EMS ICPC-2 codes and ED ICPC-2 codes were compared manually to examine whether the presenting complaint had altered. The results are presented with tables including percentages and numbers of EMS dispatch codes, EMS ICPC-2 codes and ED ICPC-2 codes, the age distribution (under/over 70 years) and areal distribution. Overall, the information of less than five patients were considered too specific and thus not relevant for this study and excluded from later analysis.

The information collected from both patient information systems were connected and the results presented, including the frequency and percentage of X-rays, CT-scans, laboratory tests and medications. The age distribution (under/over 70 years) of the patients receiving no medical treatment nor having any diagnostic test taken, and the age distribution (under/over 70 years) of the patients receiving some medical treatment or having diagnostic test taken were tabulated. The groups were compared using the Chi-squared test.

Microsoft Office Excel version 16.49 was used in the analyses of the data.

## Results

### EMS missions and non-urgent patient groups

A total of 117 EMS missions with 117 patients met the admission criteria and were included in the study. 51.3% (n = 60) of the patients were 18–69 years old, and 48.7% (n = 57) were over 70 years old (Table 1). The mean age of the whole study population was 62.8 years, with a standard deviation of 22.4 years. The mean ages in the subpopulations were 45.6 years with a standard deviation of 17.0 years (under 70) and 81.6 years with a standard deviation of 8.3 years (over 70). The ambulances had been dispatched using 11 different EMS dispatch codes, and the most common ones were “patient transport” (29.1%), “decrease in common general condition” (16.2%), “abdominal pain” (15.4%), “fall” (12.8%) and “mental disorder” (12.8%) (Table 1).

**Table 1:** Distribution of EMS missions by EMS dispatch codes, area and age-groups

EMS Missions	Total		Uusikaupunki		Loimaa		Age < 70		Age > 70	
	**n**	**%**	**n**	**%**	**n**	**%**	**n**	**%**	**n**	**%**
All EMS missions	**117**	**100**	**50**	**42.7**	**67**	**57.3**	**60**	**51.3**	**57**	**48.7**
Patient transport	34	29.1	11	32.4	23	67.6	21	61.8	13	38.2
Decrease in common general condition	19	16.2	9	47.4	10	52.6	5	26.3	14	73.7
Abdominal pain	18	15.4	9	50	9	50	10	55.6	8	44.4
Fall	15	12.8	7	46.7	8	53.3	< 5	-	14	93.3
Mental disorder	15	12.8	7	46.7	8	53.3	14	93.3	1	6.7
Other	6	5.1	< 5	-	< 5	-	< 5	-	< 5	-
Back/hip pain	5	4.3	< 5	-	< 5	-	< 5	-	< 5	-
Diarrhea/nausea	5	4.3	< 5	-	< 5	-	< 5	-	< 5	-

EMS, Emergency medical service

There were 44 different ICPC-2 codes used in the EMS phase, and the most common ones were D01 “abdominal pain/cramps general” (12.8%), P76 “depressive disorder” (12.0%), A03 “fever” (n = 10.3%), P98 “psychosis NOS/other” (5.1%) and S18 “laceration/cut” (5.1%) (Table 2). Most (85.5%) of the ICPC-2 codes remained unchanged in the ED, but there was a decrease in A03 “fever” and D01 “abdominal pain”.

**Table 2:** Distribution of ICPC-2 codes (N = 117)

	EMS		ED	
ICPC-2 Code	**n**	**%**	**n**	**%**
D01 abdominal pain	15	12.8	14	12
A03 fever	12	10.3	8	6.8
P76 depressive disorder	14	12	14	12
P98 psychosis NOS/other	6	5.1	6	5.1
S18 laceration/cut	6	5.1	6	5.1
L13 hip symptom/complaint	5	4.3	5	4.3
Other	59	50.4	64	54.7

ICPC-2, International Classification of Primary Care, 2nd edition; EMS, emergency medical service; ED, emergency deparment

### Diagnostic tests and treatments in university hospital during the night of the arrival

In 73.5% of the cases (n = 86), patients did not receive any medical treatment or had no diagnostic tests conducted (Table 3). Native X-rays were taken on 6.0% of the patients, and CT-scan were taken on 4.3% of the patients. Laboratory tests were taken from 6.9% of the patients, and 21.6% of patients received medication. Patients older than 70 years received significantly more medications and had more diagnostic tests taken than younger ones (*P =* .013).

**Table 3:** Distribution of diagnostic tests and treatments (N = 117 patients)

Tests/ treatments	n	%
X-ray	7	6
CT-scan	5	4.3
laboratory	27	23.1
medication	8	6.8
none	86	73.5
none, age > 70 years	36	41.9
any, age > 70 years	21	67.7
none, age < 70 years	50	58.1
any, age < 70 years	10	32.3

Half of the patients with A03 “fever” or D01 “abdominal pain” and almost half of the patients with R83 “respiratory infection other” (n = 20) received some medical treatment or had some diagnostic test taken (specific data not shown due to the small number of patients).

Overall, patients with ICPC-2 codes L13 “hip symptom/complaint”, P76 “depressive disorder”, P98 “psychosis NOS/other” and patients with S18 “laceration/cut” (total n = 36, 30.8%) did not receive any medication and did not have any diagnostic tests taken (specific data not shown due to the small number of patients).

## Discussion

The main results based on the 117 non-urgent EMS missions investigated in this study were: (1) the most common EMS dispatching codes were patient transfer, decrease in common general condition, abdominal pain, mental condition, and falling, (2) 73.5% of these low-acuity patients conveyed to a university hospital during nighttime by the EMS did not receive any medication or have any diagnostic tests taken before the next morning, (3) the most common presenting complaints were “hip symptom/complaint”, “depressive disorder”, “psychosis NOS/other” and “laceration/cut”, and (4) over 70 years old patients received more medical treatments and had more diagnostic tests taken during nighttime than younger patients.

It was interesting to note that more than every fourth EMS mission was a patient transport from a local hospital to the university hospital with a doctor’s referral. However, no single ICPC-2 code stood out from this patient group. Surprisingly, these patients referred by a doctor did not receive any medication nor had any diagnostic tests taken during the nighttime. This would suggest that the ED would not be the most appropriate place for these patients. However, the real reason for the conveyance can also be overcrowding of the local hospital’s wards or the end of the operating hours in the sending ED. If this is the case, conveyance is not in the best interest of the patient and does not promote patient safety. This phenomenon is interesting in many ways, as EMS units should be used for patient transports only if the patient’s condition requires an advanced level of care during the transport. Evidence shows that several non-medical factors can influence the decision to convey to the ED [31]. Thus, further research is needed to determine the root causes of patient transports during nighttime.

Previous studies indicate that patients who have self-harmed themselves might benefit from alternative pathways of care [25], and patients with psychiatric complaints can be directed to specialty care after evaluation from an advanced level paramedic [26]. However, it has also been shown that patients with a history of mental illness are more likely to recontact emergency care if non-conveyed by EMS [32]. In our material, none of the patients with mental conditions such as depression or psychosis received medication or had diagnostic tests taken during the first night at the university hospital. This supports the idea that alternative pathways of care might be beneficial to those patients. However, it is important to note that, despite the results, these patients have been under observation or might have received some treatments that were not included in the data of this study. The severity of the mental conditions remained unclear in this study design, and thus it is beyond the scope of this study to make recommendations on these specific patients.

In the study area, EMS response times in urgent missions are currently longer than the targets set by the hospital district [33]. However, developing alternative pathways of care in collaboration with primary healthcare for all low-acuity patients not needing treatment or diagnostic tests in the hospital during nighttime would release EMS resources to urgent missions and therefore affect the quality of EMS services. Still, this requires further research to ensure patient safety is not compromised, as the patients in this study have been, however, under observation in the ED.

According to our results, approximately half of the patients with “fever”, “abdominal pain”, and “respiratory infections” had laboratory tests taken in the ED and, because of that, are likely not suitable for non-conveyance in the current EMS setting without expanding paramedics’ scope of practice. Indeed, implementing new paramedic operational models has had positive experiences, for instance, in COVID-19 assessments [34]. However, it has been shown that non-conveyed patients with abdominal pain or fever are likely to be admitted to ED within 48 h after EMS contact [35]. Fever can also be an early sign of bacteremia that leads to a septic shock and early suspicion of sepsis is associated with improved survival [36]. However, with the use of EMS-suitable structured screening tools, paramedics could be able to separate septic patients from patients with less severe infections [37, 38]. This would allow non-conveyance of those patients that could be treated the next morning at a local hospital or even at home without risking patient safety. Considering the large number of patients with fever, conducting additional research is warranted in order to develop safe EMS protocols for treating these patients at home. This would not only enhance the quality of EMS services but also improve patient outcomes.

When non-conveyance is considered, the age of the patient should be taken into account. Elderly patients are more likely to contact the EMS again if not conveyed [39]. In addition, our results can be interpreted as supporting the conveyance of elderly patients because they are medicated and diagnostically tested more often than younger patients. The specific reasons why this is the case are worthy of further investigation. In addition, the patient experience of not being conveyed requires additional research [40].

## Limitations of the study

The data of this research consists of official patient records, the data collection is reproducible, and all suitable patients and EMS missions according to the admission criterion were included in the study. However, the relatively small number of patients reduces the generalizability of this study. As the conveyed low-acuity patients are quite a specific and small patient group in total, this representative sample can nevertheless be considered to be sufficient for a pilot study.

The data collection period was chosen to be the calendar year 2020 to avoid seasonal distortion and ensure the operating models were settled after the change in the operating hours of the two local hospitals in 2017. However, it should be noted that the data was collected during the COVID-19 pandemic when the number of EMS missions and ED visits decreased. This may affect the generalizability of the results in the post-pandemic period, and future studies should also include data from years unaffected by the pandemic.

The data contains patient records from people living in two different areas to avoid geographical distortion. Although the data are fundamentally comprehensive and accurate, some patients might not be recorded in the EMS patient information system due to maintenance outages. Further, ICPC-2 codes are not very specific, and one code can represent a broad spectrum of patients. Treatments registered only in text format are not included in this study. In addition, patient observation and monitoring are outside the scope of this study. Hence, further research utilizing quantitative and qualitative methods is needed to verify and expand upon the preliminary findings of this pilot study.

## Conclusions

A significant number (73.5%) of low-acuity patients conveyed by EMS to university hospital ED during nighttime do not receive any medication or have any diagnostic tests taken before the next morning. It might be feasible to consider alternative pathways of care, at least for patients with mental conditions, hip pain/complaint, or laceration/cut, as such patients are not receiving any medication or tests. Patients with abdominal pain or fever require diagnostic tests that are not currently available in EMS and therefore are not suitable for non-conveyance without expanding a paramedic’s scope of practice. Patients older than 70 years are more likely to be medicated or diagnostically tested than younger patients.

According to this pilot study, further research is needed to ensure the quality of care and patient safety when developing alternative pathways of care and expanding the paramedic’s scope of practice to increase the amount of non-conveyance EMS missions to avoid impolitic use of EMS and further, to reduce the workload of the ED.

## Data Availability

The datasets generated and analyzed during the current study are not publicly available due to the inclusion of sensitive patient information but are available from the corresponding author upon reasonable request.

## Abbreviations

ED: emergency department
EMS: emergency medical system
HEMS: helicopter emergency medical services
ICPC-2: International Classification of Primary Care, 2nd edition
